# Dehydration associates with lower urinary tract symptoms in progressive multiple sclerosis

**DOI:** 10.1111/ene.16175

**Published:** 2023-12-20

**Authors:** Stefania Kaninia, Charlotte M. Stuart, Ian Galea

**Affiliations:** ^1^ Clinical Neurosciences, Clinical & Experimental Sciences, Faculty of Medicine University of Southampton Southampton UK

**Keywords:** bladder, dehydration, multiple sclerosis, osmolality, urine

## Abstract

**Background:**

Lower urinary tract symptoms (LUTS) are common in persons with progressive multiple sclerosis (pwPMS), who may consequently limit their fluid intake. We aimed to investigate the hypothesis that LUTS associate with objective evidence of inadequate hydration status in pwPMS.

**Methods:**

In this prospective study, 55 pwPMS were studied over 2 years. A 6‐monthly first‐morning urine specimen was analysed for urinary osmolality and sodium as hydration markers. LUTS symptom severity in three categories (urgency, voiding and discomfort) was assessed and quantified using a questionnaire. Correlation between LUTS severity and hydration was assessed within subjects and between subjects, controlling for age.

**Results:**

Some 274 urine samples with accompanying LUTS data from 55 participants were analysed. Biochemical data showed the expected loss of urine‐concentrating capacity with increasing age. Inadequate hydration was observed in 47% of participants. LUTS were very common (87% reported urgency and 89% voiding symptoms). Voiding and discomfort, but not urgency severity, were correlated with hydration markers, both within and between participants.

**Conclusions:**

LUTS are very common in pwPMS, and associate with inadequate hydration. The causes and consequences of inadequate hydration in MS need further study, since (i) this will focus greater attention on LUTS management in pwPMS and (ii) dehydration has been associated with reversible cognitive dysfunction and physical underperformance.

## INTRODUCTION

Multiple sclerosis (MS) is a chronic disorder of the central nervous system and can manifest with sensory symptoms, mobility and balance difficulties, visual deficits, cognitive problems and sphincter disturbances [1]. Lower urinary tract symptoms (LUTS) are very frequent in persons with MS (pwMS). A recent meta‐analysis estimated that the prevalence of self‐reported LUTS was approximately 68% in pwMS [2]. LUTS manifest on average 6 years after the onset of MS and in 10% of pwMS these are reported at the time of the initial MS manifestation [3]. In the NARCOMS registry for pwMS, nocturia was reported to be the most troublesome LUTS, followed by urgency, frequency, incomplete bladder emptying and urge incontinence [4].

LUTS may present as problems of storage or voiding. Storage or irritative symptoms include urgency, frequency, nocturia and incontinence and can be attributed to detrusor overactivity. Voiding or obstructive symptoms include hesitancy, reduced and intermittent stream, straining to urinate, double voiding, and feeling of incomplete bladder emptying post voiding and are the result of detrusor sphincter dyssynergia or detrusor underactivity [5]. Storage and voiding symptoms frequently co‐exist and can cause variable degrees of discomfort, but the latter has not been formally studied in MS. Predisposing factors to LUTS have not been extensively studied, but it is known that LUTS prevalence increases with disease duration and the extent of brain and spinal cord lesions [6].

It is a common clinical observation that LUTS in pwMS can be disabling, limiting social engagement, leading to anxiety, and affecting quality of life. Restricting the oral intake of fluids to control LUTS seems to be common practice in pwMS. In a recent questionnaire study, nearly half of the participants reported restricting their fluid intake [7]. No association was found between total fluid intake and LUTS severity, but the study was based on subjective reporting of fluid intake and was cross‐sectional in nature. Moreover, food can contribute to 27%–36% of total water intake [8]. Hence, more objective assessment of hydration status is required.

Mild hypohydration has a negative impact on various cognitive tasks in healthy individuals [9]. Fatigue was associated with hydration status in pwMS, with higher fatigue scores in those with low hydration status [10]. Therefore, inadequate hydration may exacerbate the cognitive difficulties and fatigue experienced by pwMS. Mild dehydration can predispose to urolithiasis, constipation and urinary tract infections [11], thereby contributing to comorbidity in pwMS. In summary, studying the relationship between LUTS and hydration status is important since better management may improve cognition, physical performance and well‐being in pwMS.

Hydration status may be objectively assessed using biomarkers [11] and urine osmolality (UOsm) has been validated as such a marker [12, 13]. In this study, we objectively assessed whether and to what extent persons with progressive MS (pwPMS) restricted their hydration and whether this was related to LUTS.

## METHODS

### Study design and participants

In this prospective study, we hypothesised that LUTS and inadequate hydration are common in pwPMS, and they associate with each other. Primary outcome measures were LUTS as assessed by a questionnaire and urinary osmolality, a urine biomarker of hydration. A secondary outcome measure was urinary sodium. We followed up adults with primary progressive MS (PPMS) or secondary progressive MS (SPMS) every 6 months for 2 years. Inclusion criteria were (i) age ≥18 and ≤70 years and (ii) a diagnosis of MS. Female pwPMS who were pregnant or breast‐feeding were excluded. PPMS was defined according to the 2010 McDonald criteria [14]. SPMS was defined as sustained and steady progression in the preceding 2 years, confirmed by either an increase of at least one Expanded Disability Status Scale (EDSS) point or clinical documentation of increasing disability, where such worsening was not relapse‐driven, similar to contemporary studies [15, 16]. This study was approved by the National Research Ethics Service (12SC0176) and the authors’ institution (ERGO5562). Written informed consent was obtained from participants. Data to inform sample size calculation were not available but we considered that with 55 subjects a correlation coefficient of 0.35 will have 80% power to pick up a significant difference from 0 using a two‐sided test at the 5% significance level. The study conformed with the World Medical Association's Declaration of Helsinki.

### Urine samples and analyses

Participants were trained to collect a first‐morning midstream urine sample using a urine Monovette (Sarstedt) at home. Collection started when the research team were satisfied with technique, and samples were frozen on the same day. All samples were analysed together at the end of the study. UOsm was measured on an A_2_O Advanced Automated Osmometer (Advanced Instruments) using freezing point depression technology. Urine sodium and potassium were measured on an AU680 clinical chemistry analyzer (Beckman Coulter) using indirect potentiometry with sodium and potassium ion selective electrodes relative to a reference electrode. UOsm in first‐morning urine has been specifically validated against water intake [17]; a single first‐morning sample is more pragmatic than a 24‐h collection in a cohort study.

### Multiple Sclerosis Functional Composite

The Multiple Sclerosis Functional Composite (MSFC) [18] is a measure of MS‐related disability, providing measures of upper and lower limb function (Nine‐Hole Peg Test and Timed 25‐Foot Walk, respectively) and cognition (the Paced Auditory Serial Addition Test). *Z*‐scores were computed with reference to the baseline visit. Lower MSFC *Z*‐scores indicate more severe MS disability.

### LUTS questionnaire

A published questionnaire [19] was adapted to collect data on LUTS in pwPMS grouped into urgency, voiding and discomfort symptoms (Table 1). This questionnaire was previously employed in the setting of predicting pyuria and urinary tract infection, and had good psychometric qualities [19]. Participants were instructed to complete the questionnaire online within 6 days of the urine sample collection, based on their symptoms at the time. Responses were coded as Yes = 1 (symptom present) and No = 0 (symptom absent). The scores for urgency, voiding and discomfort symptoms were obtained by summing the coded responses. High scores in each of the three categories reflected worse symptoms experienced by the participants.

**TABLE 1:** Questionnaire on urgency, voiding and discomfort symptoms.

Question	Some	None
Urgency symptoms		
Urinary urgency (Do you have a sudden need to rush to the toilet to urinate?)	Yes	No
Urinary urge incontinence (When rushing to the toilet do you leak before you reach the toilet?)	Yes	No
Cold weather exacerbation (Does cold weather make your urgency worse?)	Yes	No
Running water urgency (Does the sound of running water make your urgency worse?)	Yes	No
Running water incontinence (Have you leaked urine on hearing the sound of running water?)	Yes	No
Latchkey urgency (Do you have urgent need to pass urine when you put the key in your front door?)	Yes	No
Latchkey incontinence (Do you leak urine when you put the key in your front door?)	Yes	No
Waking rising urgency (Do you have to rush to the toilet on waking up?)	Yes	No
Waking rise incontinence (Do you have urgency and leak urine on waking up?)	Yes	No
Anxiety fatigue aggravation (Does your urgency worsen when you are tired or anxious?)	Yes	No
Premenstrual aggravation (Does your urgency worsen prior to a period?)	Yes	No
Leaking when coughing with urgency (If you had urgency and coughed would you leak?)	Yes	No
Voiding symptoms		
Hesitancy (Is there a delay before you start to urinate?)	Yes	No
Reduced stream (Do you feel the urine stream is reduced compared to before?)	Yes	No
Intermittent stream (Do you stop and start more than once when you urinate?)	Yes	No
Straining to void (Do you have to push or strain to pass urine?)	Yes	No
Terminal dribbling (At the end of your urination do you dribble?)	Yes	No
Post‐micturition dribbling (Do you dribble urine straight after you have finished urinating?)	Yes	No
Double voiding (Do you need to sometimes go twice in a short space of time to urinate, e.g., 5 min apart)	Yes	No
Incomplete emptying (Do you feel that your bladder is not fully emptied?)	Yes	No
Discomfort symptoms		
Bladder pain on filling (Do you experience any bladder pain or discomfort when it is full?)	Yes	No
Bladder pain relieved by voiding (pain or discomfort relieved after emptying)	Yes	No
Bladder pain partially relieved by voiding (discomfort relieved slightly after emptying)	Yes	No
Bladder pain unrelieved by voids (pain or discomfort not relieved after emptying?)	Yes	No
Bladder or suprapubic pain (Do you suffer from pain in the bladder area?)	Yes	No
Loin pain (Do you suffer from pain in the kidney area?)	Yes	No
Dysuria (Do you suffer from pain during urination in the urethral area?)	Yes	No
Urethral pain (Do you suffer from pain in the urethral area?)	Yes	No
Pain or discomfort referred to genitals (Do you have pain going to the genital area?)	Yes	No
Left or right iliac fossa pain (Do you have pain in the lower part of your tummy?)	Yes	No
Pain radiating to legs (Do you have pain going down the tops of your thighs?)	Yes	No
Bladder pain during micturition (Do you have pain while passing urine?)	Yes	No
Pain after micturition (Do you have pain after urinating?)	Yes	No

### Statistics

Analysis was performed in SPSS Statistics version 28 (IBM). Data distribution was assessed using frequency histograms, Q–Q plots and the Shapiro–Wilk test. Data were normally distributed other than the EDSS. The mean UOsm approached a bimodal distribution but did not deviate significantly from a normal distribution (Shapiro–Wilks *p* = 0.122). Multivariable regression, partial correlations and zero‐order correlations were employed as appropriate. There were repeated sets of observations for each participant. Hence between‐subject analyses were conducted on means weighted for the number of observations [20] and within‐subject analyses were conducted with analysis of covariance to account for between‐subject variation [21]. A *p* < 0.05 was considered statistically significant.

## RESULTS

### Demographics, clinical and study characteristics

Urine samples and LUTS questionnaires from 55 participants were analysed in this study. Baseline demographic data and clinical characteristics of the study population are shown in Table 2. As expected for a mixed primary/secondary progressive MS population, the mean age was 55 years, with a long disease duration (mean 13 years), significant disability (median EDSS = 6.0) and there was a slight female preponderance. No MS relapses occurred during the study. We analysed 274 first‐morning urine samples from 55 participants, with accompanying LUTS questionnaire data. The mean number of observations per participant was five, with a mean interval between observations of 7.1 ± 4.4 months. The mean interval between urine sample acquisition and LUTS questionnaire completion was 2.2 ± 1.0 days.

**TABLE 2:** Baseline demographic data and clinical characteristics of the study cohort.

Parameter	Value
pwMS, *n*	55
Mean age, years (SD)	54.6 (7.7)
Male:female ratio (%)	25:30 (45.5:54.5)
Primary progressive:secondary progressive MS ratio (%)	31:24 (56.4:43.6)
Mean disease duration, years (SD)	12.7 (8.6)
Median EDSS (IQR)	6.0 (0.5)
Clean intermittent self‐catheterization, *n* (%)	4 (7)
Participants on medications for bladder control, *n* (%)	16 (29)
LUTS at baseline, *n* (%)	
Urgency	48 (87)
Voiding	49 (89)
Discomfort	26 (47)

Abbreviations: EDSS, Expanded Disability Status Scale; IQR, interquartile range; LUTS, lower urinary tract symptoms; MS, multiple sclerosis; pwMS, persons with multiple sclerosis; SD, standard deviation.

### LUTS

The frequency of LUTS of any level of severity at baseline is shown in Table 2. There was no correlation between LUTS severity and EDSS score at baseline, in keeping with the EDSS being mainly driven by ambulatory capability in this study population. The severity of voiding symptoms correlated with a worse (lower) MSFC score at baseline (Pearson's *r*: −0.30, *p* = 0.029), but urgency and discomfort symptoms did not (Pearson's *r*: −0.20 and −0.02, *p* = 0.152 and 0.893, respectively). There was no significant difference in baseline urgency or voiding severity between participants on pharmacological treatment for storage symptoms versus untreated participants (4.3 ± 3.2 vs. 3.8 ± 3.0, independent samples *t*‐test, *p* = 0.589).

### Biochemical data validation

**FIGURE 1:**
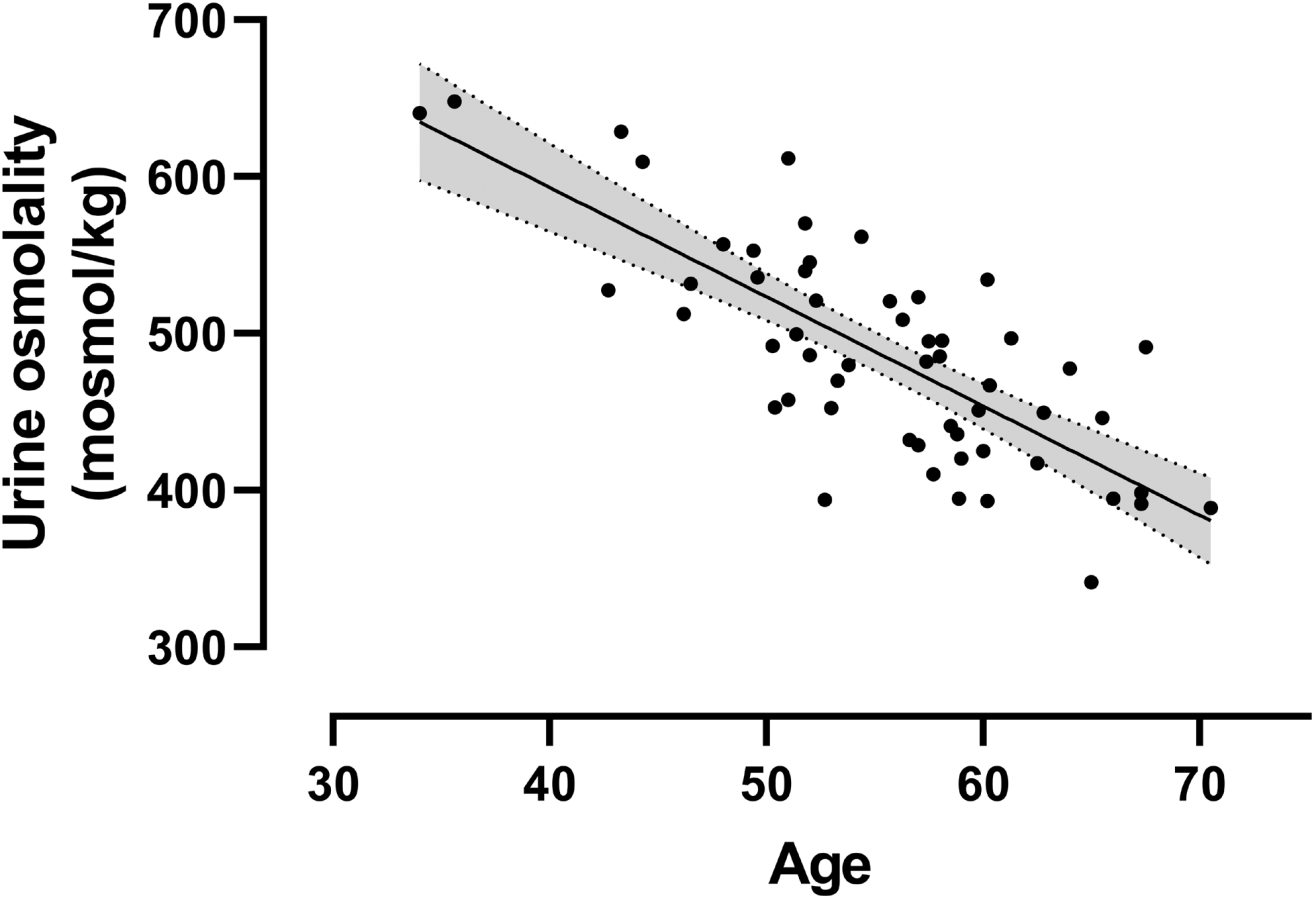
Urine osmolality (UOsm) was anti‐correlated with age (partial *r* = −0.30, *p* = 0.030). Values on *y* axis were predicted from multivariable regression of mean UOsm on mean age, gender, disease duration and baseline multiple sclerosis functional composite. Line represents regression with shaded 95% confidence interval.

It is well established that age is associated with reduced urine concentrating ability [22, 23, 24] and this fact was employed to test the validity of this study's biochemical dataset. In a multivariable regression of mean UOsm against age, gender, disease duration and baseline MSFC, age was a significant predictor, and this association was in the expected direction (partial *r*: −0.30, *p* = 0.030; Figure 1).

Another important, well‐established relationship useful to validate the biochemical data is that urinary sodium and potassium concentrations are correlated with UOsm in non‐severely dehydrated persons varying their water intake [22]. Within subjects, UOsm was significantly correlated with urinary sodium (coefficient: 0.72, *p* < 0.001) and urinary potassium (coefficient: 0.66, *p* < 0.001). Between subjects and controlling for age, mean UOsm was significantly correlated with mean urinary sodium (*r*: 0.79, *p* < 0.001) and mean urinary potassium (*r*: 0.74, *p* < 0.001).

### No evidence of severe hypohydration

In severe dehydration the renin–angiotensin–aldosterone system is activated, and the urine sodium/potassium ratio decreases as UOsm increases [25]. There was no evidence of an anti‐correlation between urinary sodium and potassium (within subject *r*: 0.32, *p* < 0.001 and between subject *r*: 0.45, *p* < 0.001). In addition, the urine Na/K ratio was not anti‐correlated with UOsm (within‐subject *p* = 0.541 and between‐subject *p* = 0.260), even within the subgroup of participants with mean UOsm > 500 mosmol/kg (*n* = 26), the European Food Safety Authority's threshold for adequate hydration [26] (within‐subject *p* = 0.814 and between‐subject *p* = 0.192). Therefore, there was no evidence of severe hypohydration in the study cohort.

### Inadequate hydration

**FIGURE 2:**
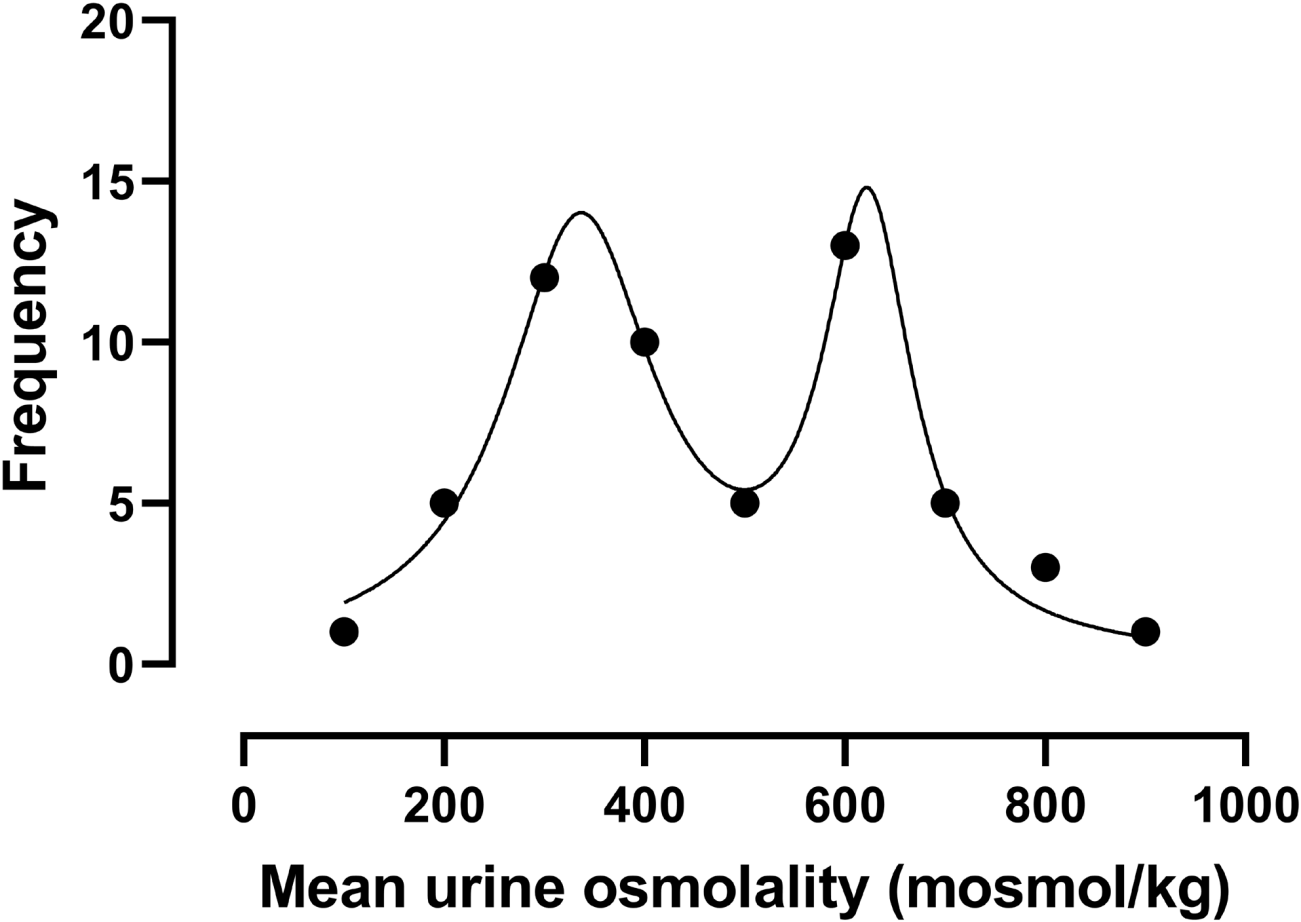
Mean urine osmolality (UOsm) approached a bimodal distribution as shown here by the frequency of the mean UOsm of each participant. Data were divided into 100 mosmol/kg‐wide bins and the frequency of each bin is represented by the dots. Values on *x*‐axis represent bin centres. Curve‐fitting used a sum of two Lorentzian model.

In order to assess the hydration status of pwPMS in this cohort, we assessed the distribution of the mean UOsm of each participant. As comparative thresholds, we used the European Food Safety Authority's threshold of UOsm < 500 mosmol/kg as an index of adequate hydration, UOsm > 800 mosmol/kg as a marker of significant hypohydration [27], and milder degrees of hypohydration between these two thresholds. Mean UOsm approached a bimodal distribution around the 500 mosmol/kg threshold (Figure 2). In half of the participants (*n* = 26, 47%) hydration was not adequate (i.e., UOsm was ≥500 mosmol/kg). The number of urine samples on either side of the 500 mosmol/kg threshold was well balanced (149 samples < 500 mosmol/kg, 125 samples ≥ 500 mosmol/kg, chi‐squared test *p* = 0.3). Three participants (5%) were significantly dehydrated with UOsm > 800 mosmol/kg.

### Correlation of LUTS severity with urinary osmolality

Participants with a mean UOsm ≥ 500 mosmol/kg, compared to those with mean UOsm < 500 mosmol/kg, had more severe voiding symptoms (mean 4.8 ± 3.6 vs. 2.6 ± 5.0, *p* < 0.001, weighted unpaired *t‐*test). The severity of urgency and discomfort symptoms was not significantly different (mean 4.1 ± 6.0 vs. 3.3 ± 7.1, *p* = 0.353 and 1.4 ± 4.5 vs. 0.6 ± 2.6, *p* = 0.109 respectively, weighted unpaired *t‐*test).

**FIGURE 3:**
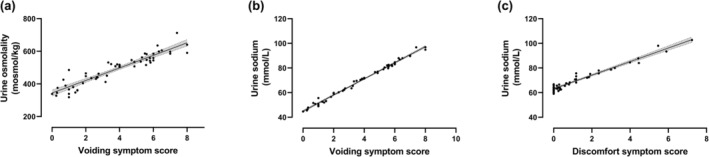
Correlation of lower urinary tract symptoms (LUTS) with urinary hydration markers. (a–c) Between subject correlations using subject means. (a) Urine osmolality (UOsm) versus voiding, (b) urine sodium versus voiding and (c) urine sodium versus discomfort. Line represents regression with shaded 95% confidence interval.

Between subjects, using UOsm as a hydration marker and correcting for age, there was evidence of inadequate hydration (higher mean UOsm) with increasing severity of voiding symptoms (partial *r*: 0.45, *p* < 0.001; Figure 3a) but not urgency or discomfort symptoms (Table 3). The multiple sampling in the study allowed us to correlate LUTS with hydration within subjects: a higher UOsm was associated with more severe voiding symptoms (partial *r*: 0.17, *p* = 0.012), but not urgency or discomfort symptoms (Table 4).

**TABLE 3:** Between‐subject correlations of lower urinary tract symptoms (LUTS) with urinary hydration biomarkers (multivariable linear regression weighted for number of observations and correcting for age).

LUTS	Urinary osmolality		Urinary sodium	
	Partial *r*	*P* value	Partial *r*	*P* value
Urgency	0.11	0.439	0.18	0.190
Voiding	0.45	<0.001	0.47	<0.001
Discomfort	0.16	0.264	0.28	0.039

**TABLE 4:** Within‐subject correlations of lower urinary tract symptoms (LUTS) with urinary hydration biomarkers (analysis of covariance, correcting for age and between‐subject variation).

LUTS	Urinary osmolality		Urinary sodium	
	Partial *r*	*P* value	Partial *r*	*P* value
Urgency	0.04	0.540	0.05	0.455
Voiding	0.17	0.012	0.15	0.026
Discomfort	−0.12	0.123	−0.17	0.024

### Correlation of LUTS severity with urinary sodium

Between subjects, using urinary sodium as a hydration marker and correcting for age, there was evidence of inadequate hydration (higher mean urinary sodium) with more severe voiding symptoms (partial *r*: 0.47, *p* < 0.001; Figure 3b) and discomfort symptoms (partial *r*: 0.28, *p* = 0.039; Figure 3c), but not urgency symptoms (Table 3). Within subjects, urinary sodium was higher with more severe voiding symptoms (partial *r*: 0.15, *p* = 0.026), lower with more severe discomfort symptoms (partial *r*: −0.17, *p* = 0.024), and unrelated to urgency symptoms (Table 4).

## DISCUSSION

In this study of 55 participants with progressive MS, inadequate hydration was present in half of the pwPMS population studied and was proportional to voiding and discomfort symptom severity. Whether pwPMS with emptying disorders limit hydration was not clear, and this study suggests that this is the case. This was also observed within subjects, so that when individual participants' voiding symptoms were worse, they appeared to fluid restrict, resulting in more concentrated urine. There was no evidence supporting a relationship of inadequate hydration with more severe urgency symptoms. Therefore, these data show that inadequate hydration is common in pwPMS who experience more severe voiding symptoms, most likely because individual pwPMS decrease their fluid intake to manage these symptoms. Conversely, urgency symptoms did not significantly affect hydration practices, possibly because participants with more severe urgency symptoms tended to reduce their oral intake of fluids on specific occasions (e.g., before leaving their house or while outdoors) and compensating at other times (e.g., when indoors), such that there was no overall impact on urine hydration markers.

While it is known that pwMS fluid restrict to control LUTS [7], this study provides the first objective evidence of a physiological effect, linking LUTS with inadequate hydration. Understanding practices that pwMS employ for the management of LUTS is important to address inadequate hydration and downstream effects including cognition, physical performance, well‐being and potential complications such as urolithiasis.

### Strengths

This study has several strengths. Biochemical data were internally validated. Another major strength was the multiple sampling, so that the assessment was representative over a period of time, and less prone to day‐to‐day variability. This is important since both LUTS and hydration can vary and a single cross‐sectional assessment would be highly susceptible to this variability. The multiple sampling also enabled the study of within‐subject relationships between LUTS and hydration status, in addition to between‐subject correlations. The participants were in the progressive phase of the condition, and none had relapses, thereby minimising fluctuations. The choice of hydration marker was considered at length. Chronic hydration status is best assessed with UOsm, rather than serum osmolality; the latter is more appropriate for the assessment of acute hydration status [13]. Plasma osmolality is not sensitive to changes in water intake and mild hypohydration, due to physiological adaptations [17]. Another advantage of employing urine biomarkers is that the collection of urine samples is non‐invasive, low cost and can be carried out in the domiciliary setting. Finally, first‐morning urine substitutes well for 24‐h UOsm. [17].

### Limitations

One of the limitations of our study was that the participants were in the progressive phase of MS, therefore the results cannot be generalised to the entire MS population. Sample size was not sufficiently large to study PPMS and SPMS separately. We aimed to use questions from a published questionnaire to assess LUTS, but this was not validated for pwMS; and while urgency and urge incontinence were used as indicators of bladder overactivity, the questionnaire did not cover the symptoms of nocturia and frequency [19]. It might have been better to use questionnaires which have been validated in patients with neurogenic bladder such as the Neurogenic Bladder Symptom Score [28], the Urinary Symptom Questionnaire [29] or the Urinary Symptom Profile [30, 31]. LUTS can also occur secondary to other pathologies, such as benign prostatic hyperplasia, and participants were not screened for this and other urological conditions. Urinary tract infection diagnoses were not ascertained, nor was constipation assessed, yet these may have affected the association between LUTS and hydration, since it is generally recommended to increase fluid intake during periods of infection or constipation. We did not collect information on medications which could affect fluid intake and UOsm (e.g., desmopression, antidepressants or antihypertensive medications). pwMS commonly increase the intake of caffeinated drinks to overcome fatigue, and this may exacerbate LUTS [7]. In addition, the use of antimuscarinic drugs for the management of storage dysfunction may exacerbate voiding symptoms [6], although such a relationship was not observed in this cohort.

### Future directions

LUTS were categorised into types, an approach which has been employed before [19]. Urgency and voiding symptoms were highly prevalent in this study cohort, as expected. We were interested to find that 47% of MS patients were describing discomfort. In an exploratory analysis to determine the relative contribution of underactive or overactive LUTS to discomfort, partial correlations were obtained from a between‐subject multivariable linear regression of discomfort on urgency and voiding. Discomfort could be explained by voiding symptoms (partial *r*: 0.27, *p* = 0.049) but not by urgency symptoms (partial *r*: 0.14, *p* = 0.328). The correlations suggest that this discomfort was linked to problems with voiding, and not linked to other bladder symptoms. It is possible that a component of this discomfort is generated by the sensation of fullness in the suprapubic area due to a chronically partially full bladder. Although voiding contributes to this discomfort, it only explained a small proportion of variance in discomfort (7%, partial *r*‐squared), in keeping with the fact that discomfort in the suprapubic, loin and groin areas may have several other causes in the setting of MS (such as pain of neuropathic origin, constipation or musculoskeletal issues). Additional work is needed to investigate this further.

Smith et al. studied a cohort of 54 female pwMS and found that lesion location was related to the severity of LUTS [4]. The neural circuitry controlling micturition is very complex and involves various cortical, limbic and brainstem areas [32], and some of these regions are very close to centres involved in thirst [33]. Hence it is possible that lesions bridging both micturition and thirst areas drove the association between LUTS and inadequate hydration. However, this is somewhat unlikely since the association was restricted to voiding symptoms. A future direction would be to study the location of demyelinating lesions in close proximity to thirst and micturition centres, LUTS and hydration.

## CONCLUSIONS

In summary, while the relationship between LUTS and hydration status is complex, this study provides evidence that voiding symptoms are associated with inadequate hydration in progressive MS. This suggests that fluid‐restricting behaviour, which is common practice in this population in response to LUTS, is associated with measurable changes in hydration status. Inadequate hydration potentially leads to cognitive and other functional consequences, decreases quality of life and increases morbidity in pwMS. Improved management of LUTS would help pwMS maintain adequate hydration and potentially improve their well‐being, but further study of this field is required.

## AUTHOR CONTRIBUTIONS

**Stefania Kaninia:** Investigation, data curation, formal analysis, writing—original draft, writing—review & editing. **Charlotte M. Stuart:** Investigation, data curation, writing—review & editing. **Ian Galea:** Conceptualisation, methodology, formal analysis, writing—review & editing, visualisation, supervision, project administration, funding acquisition.

## FUNDING INFORMATION

MS Society (Grant number 996).

## CONFLICT OF INTEREST STATEMENT

No disclosures relevant to the article.

## Data Availability

Anonymised data will be made available on reasonable request from any qualified investigator, in keeping with ethical and institutional approvals and policies.
